# Targeted Skipping of Human Dystrophin Exons in Transgenic Mouse Model Systemically for Antisense Drug Development

**DOI:** 10.1371/journal.pone.0019906

**Published:** 2011-05-17

**Authors:** Bo Wu, Ehsan Benrashid, Peijuan Lu, Caryn Cloer, Allen Zillmer, Mona Shaban, Qi Long Lu

**Affiliations:** McColl-Lockwood Laboratory for Muscular Dystrophy Research, Neuromuscular/ALS Center, Department of Neurology, Carolinas Medical Center, Charlotte, North Carolina, United States of America; University Hospital Vall d'Hebron, Spain

## Abstract

Antisense therapy has recently been demonstrated with great potential for targeted exon skipping and restoration of dystrophin production in cultured muscle cells and in muscles of Duchenne Muscular Dystrophy (DMD) patients. Therapeutic values of exon skipping critically depend on efficacy of the drugs, antisense oligomers (AOs). However, no animal model has been established to test AO targeting human dystrophin exon in vivo systemically. In this study, we applied Vivo-Morpholino to the hDMD/*mdx* mouse, a transgenic model carrying the full-length human dystrophin gene with *mdx* background, and achieved for the first time more than 70% efficiency of targeted human dystrophin exon skipping in vivo systemically. We also established a GFP-reporter myoblast culture to screen AOs targeting human dystrophin exon 50. Antisense efficiency for most AOs is consistent between the reporter cells, human myoblasts and in the hDMD/*mdx* mice in vivo. However, variation in efficiency was also clearly observed. A combination of in vitro cell culture and a Vivo-Morpholino based evaluation in vivo systemically in the hDMD/*mdx* mice therefore may represent a prudent approach for selecting AO drug and to meet the regulatory requirement.

## Introduction

Antisense therapy has been shown promising for the treatment of Duchenne muscular dystrophy (DMD) [1]–[16]. DMD, affecting approximately 1 of 3500 new-born boys, is a severe x-linked muscle wasting disease caused by nonsense and out of frame mutations of the human DMD gene [17]. The dystrophin gene is one of the largest genes in the human genome containing 79 exons spanning more than 2.3 million base pairs. The muscle form of dystrophin consists of four structural domains: amino terminal, rod, cysteine-rich, and carboxy terminal. The rod domain where most DMD mutations occur spans more than half of the length of the protein with limited functions. AO therapy is applicable to nearly all types of mutations occurring in this rod region and is predicted to be able to transform more than 60% DMD to near-normal phenotypes or milder Becker muscular dystrophies [18]–[20]. The potential therapeutic effect of AOs was initially demonstrated in dystrophic *mdx* muscle cells that harbor a nonsense point mutation in the exon 23, resulting in the lack of dystrophin expression [1]. 2'O methyl phosphorothioate (2OMePS) AOs induced functional amount of dystrophin expression in tibialis anterior (TA) muscles by local injection and further showed systemic effect in body-wide muscles [4], [5]. Phosphorodiamidate morpholinos (PMO) have been reported with higher efficiency and functional levels of dystrophin production in body-wide skeletal muscles in *mdx* mice and dystrophic dog [6], [10]. More recently, we and others demonstrated that arginine-rich peptides and dendrimeric octaguanidine conjugated morpholino (PPMO and Vivo-PMO) are able to restore the reading frame and produce almost normal levels of dystrophin protein in body-wide skeletal and cardiac muscles in the *mdx* mice [7], [8]. Repeated injections of PPMO and Vivo-PMO can maintain the levels of dystrophin expression for up to 3 months without obvious toxicity. The applicability of AO therapy has now been indicated in Phase I/II clinical trials with both 2OMePS AOs and PMO targeting human dystrophin exon 51 in DMD patients [13], [16]. More recently, systemic treatment of DMD patients with PMO was reported to induce detectable amount of dystrophin protein in sampled skeletal muscles of all patients, although considerable variation in skipping efficiency was also clearly observed within the trial population. Patients after systemic administration of 2OMePS AO (PRO051) targeting human dystrophin exon 51 have been reported with improvement in muscle function [16].

AO-mediated exon skipping is a RNA-sequence-specific therapy. Optimal AO sequences require to be identified by screening. Currently both human myoblasts and fibroblasts from normal individual and DMD patients have been used for identifying AOs with efficacy to specifically skip the targeted exons [2], [11], [12], [21]–[24]. While the AOs being used in current clinical trials were largely selected through human myoblast cultures, the degree of relevance for each selected AO targeting many other dystrophin exons using such cell cultures to exon skipping in human in vivo remains to be determined. While most AOs selected in cell culture were reported with expected exon skipping efficiency in vivo, discrepancy in skipping efficiency between in cell culture and in vivo has been reported in the selection of AOs targeting dog dystrophin exon [10]. Thus, selected AO through myoblast cultures only may not be sufficient for the AO drug development. To overcome the potential difference in exon skipping efficiency and specificity between myoblasts in vitro and myofibers in vivo, ex-vivo muscle culture has been explored to verify the efficacy of selected AOs. However, muscle fibers degrade in culture within 2 days and exon skipping effect occurs most prominently after 5 days. Thus the exon skipping observed in such culture is in fact the effect of AOs on fibroblasts and myoblasts grew out from the muscle tissue plants [9]. The lack of reliable systems for screening AO targeting many human dystrophin exons, especially the lack of animal models for establishing systemic efficacy poses a challenge for AO drug development. Efforts have been made to establish animal models for in vivo testing of AO targeting human dystrophin exons [25], [26]. Most noticeable was the creation of the humanized (h)DMD mouse containing a complete copy of the human DMD gene integrated in chromosome 5 [25]. However, the expression of the normal human dystrophin lends the mouse model phenotypically normal even having the *mdx* background. This has prevented the animal model from being effective for testing AOs with the most widely used chemistries including 2OMePS AOs and PMO. This is because effective delivery of these oligos depends on leaky membrane in dystrophic muscle, thus cannot be achieved in normal muscles.

In this study, we investigated the value of a GFP reporter based myoblast culture system for selection of AOs targeting human dystrophin exon in combination with human myoblast cultures. Most AO showed similar efficiencies in both the GFP reporter myoblasts and human myoblasts with the highly effective AO for skipping human dystrophin exon 50 being identified in both cell types. The GFP reporter provided most consistent results with signals readily quantifiable for direct comparison of exon skipping efficiency. However, variation in relative efficiency between cell cultures and in vivo was observed with different AOs. Examination of selected AOs as Vivo-PMO revealed high levels of human dystrophin exon 50 skipping in body-wide muscles including cardiac muscle in the hDMD/*mdx* mice, suggesting that hDMD/*mdx* mouse is a highly valuable model for demonstrating systemic efficacy of selected AO targeting human dystrophin exons. We conclude that a multi-model screening system combining the use of cell cultures in vitro and the hDMD/*mdx* mouse in vivo could reduce potential risk of variation in efficiency between cell culture and in vivo and represents a more systematic approach for identifying AOs of high efficacy, thus better suited to meet the regulatory requirement for AO drug development.

## Materials and Methods

### Vector constructions and cell culture

The construction of the human dystrophin exon 50/GFP reporter vector was based on the procedure reported previously [27]–[30]. The human dystrophin exon 50 flanked by 600 bps of its respective intronic sequences (intron 49 on the 5′ side and intron 50 on the 3′ side) was amplified from DNA of human myoblasts. The sequence was inserted into the middle of the β-globin intron sequence which was placed inside the coding sequence of GFP gene under the control of actin promotor. C2C12 mouse myoblasts were transfected with the vector by electroporation and the transfected cells were selected by geneticin (G418). The transfected cells (C2C12hE50) were then maintained in high-glucose Dulbecco's modified Eagle's medium (DMEM, Gibco, Carlsbad, CA) with 10% heat inactivated fetal bovine serum (Gibco, Carlsbad, CA) and 1% penicillin/streptomycin at 37°C with 5% CO_2_. Normal human myoblasts were cultured in skeletal muscle growth medium (Cell applications Inc, San Diego, CA). DMD patient-derived skin fibroblasts were maintained in RPMI (Gibco, Carlsbad, CA), 10% heat inactivated fetal bovine serum, 2% L-glutamine, and 1% penicillin/streptomycin. For PMO delivery to the cells, Endo porter (Genetools, Philomath, OR) was used according to the manufacture's instruction. For 2OMePS AO delivery, Lipofectamine (invitrogen, Carlsbad, CA) was used according to the manufacture's instruction.

### Visualization of GFP by fluorescence microscopy and flow cytometric analysis

Cells, 48 hours after the AO treatment, were visualized using an Olympus IX71 inverted fluorescent microscope. Digital images of both Fluorescence and transmit light were taken using the Olympus DP Controller and DP Manager software. Percentage of GFP positive cells was obtained by counting the GFP positive cells under fluorescence light and total number of cells under transmit light (500 cells in the same fields) before the cells were subjected to flow cytometry analysis. Then, cells were examined using flow cytometry to quantitatively gauge the GFP expression level. Cells were washed with PBS (1×) and released from culture vessel with 0.05% Trypsin-EDTA, neutralized by FBS, pelleted by centrifugation and then re-suspended in 1 ml PBS. Samples were run on a Calibur flow cytometer (BD, Franklin Lakes, NJ). 2×10^4^ cells were counted and analyzed with CellQuest Pro (BD, Franklin Lakes, NJ) software package.

### RT-PCR and Nested-PCR analysis for cell cultures

For RT-PCR Analysis, cells were initially washed twice with PBS, and RNA was extracted with TriZol reagent (Invitrogene, Carlsbad, CA) according to manufacturer's instructions. RNA was stored at −80°C for later use. RT-PCR was performed with RT-PCR Master Mix (USB, Cleveland, Ohio) to amplify the sequence of interest. 100 ng of template RNA was used for each 25 µl RT-PCR reaction. The primer sequences for the RT-PCR of C2C12 hDysE50 were eGFPF: 5′ – ATGGTGAGCAAGGGCGAGGAGCTG – 3′; eGFPR: 5′ – CTGCACGCCGTAGGTCAGGGTGGT – 3′. The conditions were 43°C for 15 minutes, 94°C for 2 minutes, then cycled 30 times at 94°C for 30 seconds, 65°C for 30 seconds, and 68°C for 1 minute. The primer sequences for the RT-PCR of normal human myoblasts and DMD patient-derived fibroblasts were Ex47/48F3, 5′ – GTGGATAAAGGTTTCCAGAGC – 3′ and Ex53/52R3, 5′ – GAATTCTTTCAATTCGATCCGTA – 3′. The conditions were 43°C for 15 minutes, 94°C for 2 minutes, then cycled 30 times at 94°C for 30 seconds, 55°C for 30 seconds, 68°C for 1 minute, and 68°C for 5 minutes. The primer sequences for the Nested-PCR of normal human myoblasts and DMD patient-derived skin fibroblasts were Ex48F2, 5′ – TCTGCTGCTGTGGTTATCTC – 3′ Ex52R2, 5′ – TTTTGGGCAGCGGTAATGAG – 3′. The conditions were 94°C for 2 minutes, then cycled 30 times at 94°C for 30 seconds, 55°C for 30 seconds, 72°C for 1 minute, and 72°C for 5 minutes. The products were examined by electrophoresis on a 2% agarose gel. The intensity of 2 bands representing transcripts with and without targeted exon skipping was measured with the NIH ImageJ 1.42. The total measurement of the 2 bands was considered as 100%. 50% skipping efficiency is defined as the signal intensity of the band representing transcripts with targeted exon skipping equal to that of the band representing transcripts without targeted exon skipping.

### Animals, oligonucleotides and in vivo delivery methods and Ethical statement

This study was carried out in strict accordance with the recommendations in the Guide for the Care and Use of Laboratory Animals of the National Institutes of Health. The protocol was approved by the Committee on the Ethics of Animal Experiments of IACUC Carolinas Medical Center (Breeding protocol: 01-08-06A; Experimental protocol: 01-08-07A). All injection was performed under isoflurane anesthesia, and all efforts were made to minimize suffering.

Five C57, mdx, hDMD/mdx mice aged 5–7 weeks were used for each group unless specified otherwise [25]. The three of PMOs conjugated with a dendrimeric octaguanidine (Vivo-hE50AO23PMO, Vivo-hE50AO5PMO, Vivo-hE50AO12PMO) for targeting exon 50 of the human dystrophin gene, and Vivo-PMOE23 (+7–18) against the boundary sequences of the exon 23 and intron 23 of mouse dystrophin gene were used. Vivo-PMOs were prepared in 40 or 100 µl saline and administrated by intramuscular or retraorbital injections.

Mice were killed at desired time points, and muscles were snap-frozen in liquid nitrogen-cooled isopentane and stored at −80°C.

### Antibodies and Immunohistochemistry

Sections of 6 µm were cut from at least two–thirds of muscle length of TA, quadriceps, biceps, and gastrocnemius at 100 µm intervals and at least 10 levels from all other muscles including heart, diaphragm, intercostals, and abdominal muscles at 100 µm intervals. The intervening muscle sections were collected either for RT-PCR analysis. Sections were also stained with hematoxylin and eosin for histological assessment.

### RNA Extraction and RT-PCR for mice muscles

Collected Sections were homogenized in TRIzol (Invitrogene, Carlsbad, CA) by using an Ultra-Turrax homogenizer (Janke and Kunkel, Staufen, Germany). RT-PCR for testing mouse exon 23 skipping was done as described previously [7]. Total RNA was then extracted, and 100 ng of RNA template was used for a 50 µl RT-PCR with RT-PCR Master Mix (USB, Cleveland, Ohio). The primer sequences for testing hE50 skipping were Ex47/48F3, and Ex53/52R3 (for sequences see above) to amplify mRNA from exons 47 to 53. A total of 30 cycles were carried out for the RT-PCR. Bands with the expected size for the transcript with hE50 deleted were extracted and sequenced. The intensity of the bands was measured with the NIH ImageJ 1.42 and percentage of exon skipping was calculated with the intensity of the 2 bands representing both unskipped and skipped exons as 100%.

### Measurement of serum creatine kinase and other components

Mouse blood was taken immediately after cervical dislocation and centrifuged at 1500 rpm for 3 min. Serum was separated and stored at −80°C. The level of serum components was determined by Charles Riverside Laboratories.

## Results

### Targeting human dystrophin exon 50 in C2C12/GFP reporter myoblasts

**Figure 1:**
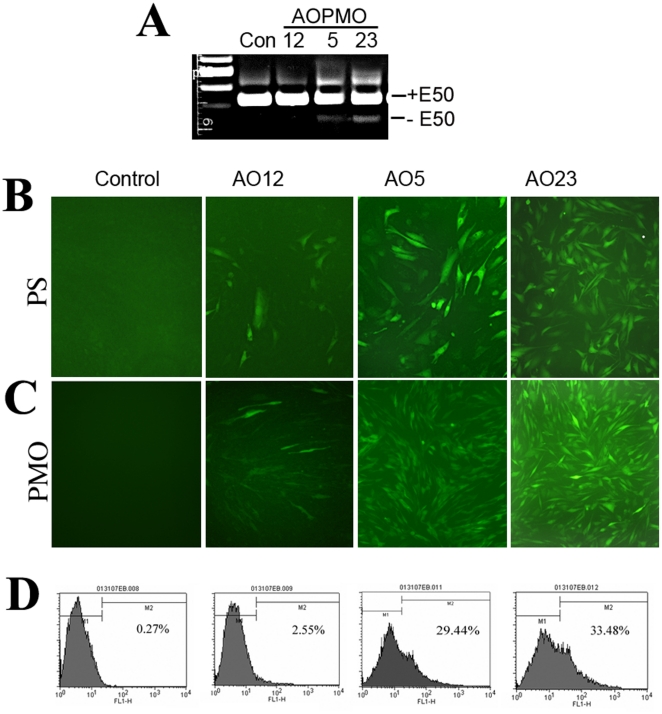
AOs target human dystrophin exon 50 in C2C12hE50 GFP reporter myoblasts 48 hours after treatment. (A). RT-PCR for the detection of GFP/hE50 mRNA in the C2C12hE50 cells. Left lane is the 100bp size marker. Con, Control sample without PMO treatment; 12, 5, 23 are the hE50AOPMOs. The bands marked with +E50 representing normal dystrophin mRNA containing E50; The bands marked with −E50, representing dystrophin mRNA with hE50 skipped. The band representing hE50 skipping was most strongly detected in the cells treated with hE50AO23PMO. (B) and (C) cells treated with the hE50AOPS and hE50AOPMO respectively. (D). FACS analysis for the GFP positive cells treated with the 3 hE50AOPMOs and the control (without AO treatment).

To identify potent AOs for targeted skipping of human dystrophin exon 50 (hE50), we synthesized a total of 25 AOs as 2OMePS (referred as hE50AO#PS) to cover more than two thirds of the hE50 and the two flanking intron sequences. Two exonic splicing enhancer (ESE) sequences (gaccacua and uacuuc) with SC35 score of 5.17 and SRp55 score of 3.22 determined by the program of ESE Finder (version 2, http://rulai.cshl.edu/tools/ESE) were also covered by two AOs, hE50AO14PS and hE50AO16PS (Table 1). The hE50AO12PS AO previously reported to be effective for hE50 skipping in human myoblasts was also included [11]. As described previously, a vector expressing GFP and the hE50 was created [30]. The GFP gene in the reporter construct is split into 2 fragments (exons) by the insertion of the human β-globulin intron sequence which is spliced out during the transcription process. The insertion of hE50 (including its two flanking intronic sequences) disrupts the reading frame of the GFP, resulting in no GFP protein expression. Effective antisense oligonucleotides targeting the hE50 will be able to remove the dystrophin exon and restore the GFP reading frame. The selected C2C12 myoblast clone transfected with the GFP/hE50 vector expressed high levels of GFP/hE50 chimeric mRNA (termed C2C12hE50), but with no detectable transcripts with hE50 skipped (Figure 1A, Control lane). Thus the cell line provides the high potential to distinguish the efficiency of exon skipping. Consistently, leaky GFP expression was barely detectable (in less than 0.2% cells with weak GFP signal detected under fluorescence microscope) (Figure 1B and C left panels). Thus, the levels of GFP expression represent the degrees of exon skipping efficiency by individual AO. All the 2OMePS oligomers were examined with the C2C12hE50 cells initially at the concentration of 1 µM. Most of the oligomers did not enhance the GFP expression at this dose (Table 1). Significant increase in the levels of GFP expression was observed in the cells treated with hE50AO12PS, hE50AO5PS and hE50AO23PS, at the rates up to 8%, 21% and 28% respectively in repeated experiments (Table 1 and Figure 1B).

**Table 1:** List of AO compounds, the sequence and size of AOs and the summary of their exon skipping effect.

AON	Target	2′OMe PS AON Sequence	L	GFP	hM	DMD
hE50 AO1PS	−24+8	5′–AACUUCCUCUUUAACAGAAAAGCAUACACAUU–3′	32 bp	-	-	N/D
hE50 AO2PS	−19−1	5′–CUUUAACAGAAAAGCAUAC–3′	19 bp	-	-	N/D
hE50 AO3PS	−19+1	5′–UCUUUAACAGAAAAGCAUAC–3′	20 bp	-	-	N/D
hE50 AO4PS	−19+3	5′–CCUCUUUAACAGAAAAGCAUAC–3′	22 bp	4%	3%	N/D
hE50 AO5PS	−19+8	5′–AACUUCCUCUUUAACAGAAAAGCAUAC–3′	27 bp	21%	29%	N/D
hE50 AO6PS	−19+13	5′–CUUCUAACUUCCUCUUUAACAGAAAAGCAUAC–3′	32 bp	3%	<1%	N/D
hE50 AO7PS	−13+3	5′–CCUCUUUAACAGAAAA–3′	16 bp	-	-	N/D
hE50 AO8PS	−14+8	5′–AACUUCCUCUUUAACAGAAAAG–3′	22 bp	-	-	N/D
hE50 AO9PS	−9+8	5′–AACUUCCUCUUUAACAG–3′	17 bp	-	-	N/D
hE50 AO10PS	−9+21	5′–GCUCAGAUCUUCUAACUUCCUCUUUAACAG-3′	30 bp	-	-	N/D
hE50 AO11PS	−8+8	5′–AACUUCCUCUUUAACA–3′	16 bp	-	-	N/D
hE50 AO12PS	+2+30	5′–CCACUCAGAGCUCAGAUCUUCUAACUUCC–3′	29 bp	8%	25%	N/D
hE50 AO13PS	+11+27	5′–CUCAGAGCUCAGAUCUU–3′	17 bp	2%	4%	N/D
hE50 AO14PS	+42+58	5′–GCUCUUGAAGUAAACGG–3′	17 bp	-	-	N/D
hE50 AO15PS	+85+102	5′–AAUAGUGGUCAGUCCAGG–3′	18 bp	-	-	N/D
hE50 AO16PS	+92−5	5′–CUUACAGGCUCCAAUAGUGGUCA–3′	23 bp	-	-	N/D
hE50 AO17PS	+92−10	5′–GUAUACUUACAGGCUCCAAUAGUGGUCA–3′	28 bp	-	-	N/D
hE50 AO18PS	+94−14	5′–UCCAGUAUACUUACAGGCUCCAAUAGUGGU–3′	30 bp	-	-	N/D
hE50 AO19PS	+97−5	5′–CUUACAGGCUCCAAUAGU–3′	18 bp	3%	-	N/D
hE50 AO20PS	+97−10	5′–GUAUACUUACAGGCUCCAAUAGU–3′	23 bp	-	-	N/D
hE50 AO21PS	+97−14	5′–UCCAGUAUACUUACAGGCUCCAAUAGU–3′	27 bp	1%	-	N/D
hE50 AO22PS	+102−14	5′–UCCAGUAUACUUACAGGCUCCA–3′	22 bp	3%	<1%	N/D
hE50 AO23PS	+103−18	5′–GGGAUCCAGUAUACUUACAGGCUCC–3′	25 bp	28%	65%	N/D
hE50 AO24PS	+105−14	5′–UCCAGUAUACUUACAGGCU–3′	19 bp	6%	-	N/D
hE50 AO25PS	+109−14	5′–UCCAGUAUACUUACA–3′	15 bp	-	-	N/D

AON	Target	PMO AON Sequence	L	GFP	hM	DMD
hE50AO5PMO	−19+8	5′–AACTTCCTCTTTAACAGAAAAGCATAC–3′	27 bp	29±2%*	28±3%*	34±3%*
hE50AO12PMO	+2+30	5′–CCACTCAGAGCTCAGATCTTCTAACTTCC–3′	29 bp	9±6%	45±5%*	25±4%*
hE50AO14PMO	+42+58	5′–GCTCTTGAAGTAAACGG–3′	17 bp	-	-	-
hE50AO23PMO	+103−18	5′–GGGATCCAGTATACTTACAGGCTCC–3′	25 bp	40±6%*	85±6%*	62±5%*
hE50AO26PMO	+7+31	5′–TCCACTCAGAGCTCAGATCTTCTAA–3′	25 bp	-	-	-
hE50AO27PMO	+94−9	5′–TATACTTACAGGCTCCAATAGTGGT–3′	25 bp	2±1%	-	-
hE50AO28PMO	+98−16	5′–GATCCAGTATACTTACAGGCTCCAATAG–3′	28 bp	4±1%	5±2%	6±2%

PS, 2OMePS AO. − and + within the Target column indicate the intron and exon sequences respectively; the numbers represent the first and last nucleotides of the oligonucleotide sequences. Percentage of GFP positive cells with 2OMePS AOs is the average from the flow cytometry analysis. Results from PMO treatment are presented as mean values ± SE with statistical analysis (ANOVA) since a completed set of exon skipping efficiency analysis including the in vivo test was conducted with those PMOs.

* *P*<0.01 was obtained by comparison of the groups with the hE50AO28PMO groups (n = 3). GFP, GFP reporter C2C12hE50 cells; hM, normal human myoblasts. DMD, DMD patient-derived skin fibroblasts with exon 51 deletion. Scores: -, no positive signal above background is observed for either GFP expression or signals representing mRNA with exon 50 skipping when compared to the samples of negative controls. Cells were treated with 1 µM AOs. N/D. Muscles of hDMD/*mdx* mice were treated with 10 µg Vivi-PMO.

To examine the potential variation in targeting efficiency with different chemistries, we synthesized and tested 7 AO sequences including the 3 most effective ones as PMOs (referred as hE50AO#PMO) at 1 µM concentration and the GFP expression was examined 48 hours after treatment (Table 1). The efficiency of hE50 skipping with the PMO chemistries was consistent to the 2OMePS oligomers of the same sequences and the highest efficiency was also obtained in the cells treated with hE50AO12PMO, hE50AO5PMO, hE50AO23PMO, hE50AO13PMO, hE50AO6PMO and hE50AO24PMO. Cells treated with hE50AO23PMO produced up to 40% of GFP positive cells in repeated experiments (3 times) (Figure 1C). The signal intensity and percentage of positive cells were generally higher with PMO than with 2OMePS of same sequence at the same dose (Figure 1B, C and Table 1). The inductions of GFP expression by the PMOs were further confirmed by flow cytometry analysis. As illustrated in the figure 1D, less than 1% GFP positive cells was recorded in the control untreated C2C12hE50 cells, but up to 29.44% and 33.48% in the cells treated with 1 µM concentration of hE50AO5PMO and hE50AO23PMO, respectively. Expected removal of the hE50 was confirmed by RT-PCR (Figure 1A).

### Screening antisense oligonucleotide targeting human dystrophin exon 50 in normal human myoblasts

**Figure 2:**
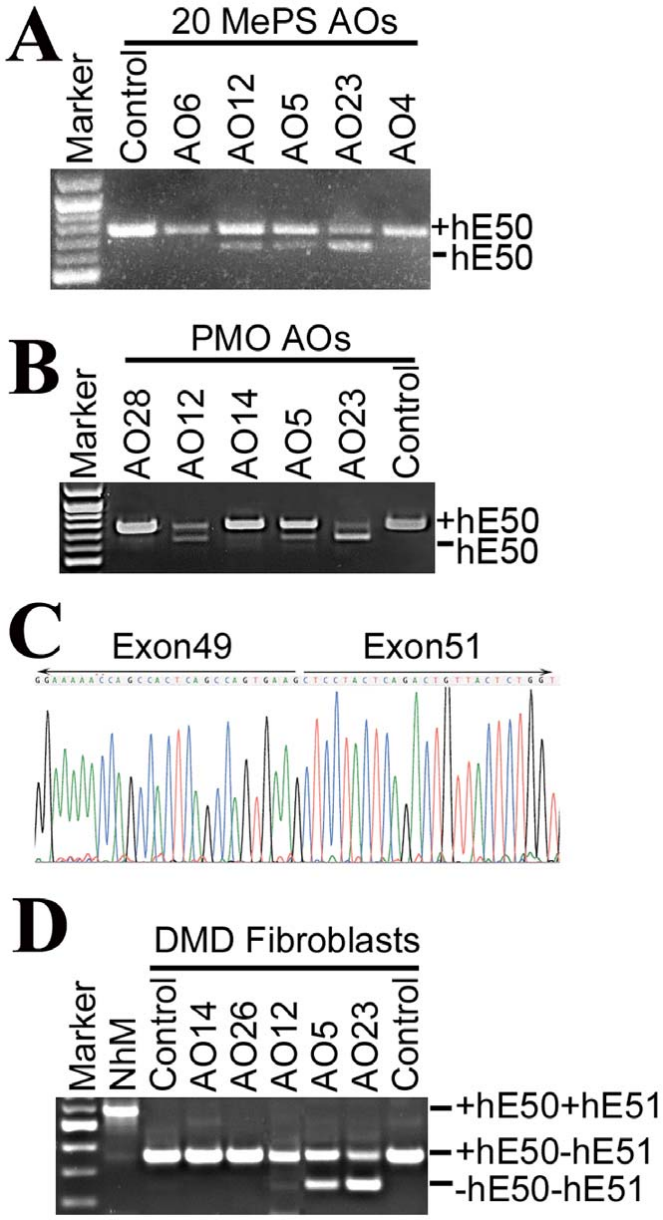
RT-PCR and Nested-PCR for the detection of hE50 skipping in the normal human myoblasts and the DMD-derived skin fibroblasts. (A). Samples from the normal human myoblasts cells treated with 2OMePS AOs; (B). Samples from the normal human myoblasts cells treated with PMOs. +hE50 representing normal dystrophin mRNA containing hE50; −hE50, representing dystrophin mRNA with hE50 skipped. Left lane is the 100 bp size marker; (C). A sequence showing skipping of the hE50 from the PCR product detected as –hE50 in the hE50AO24PMO treated normal human myoblasts cells in (B). (D). The bands marked with +hE50+hE51 representing normal human dystrophin mRNA (NhM) from normal human myoblasts; the bands marked with +hE50−hE51 representing mRNA from the skin fibroblasts of a DMD patient with exon 51 deletion; the bands marked with −hE50 and –hE51 representing mRNA from the skin fibroblasts with exon 50 skipped (both exons are absent). Control, without AO treatment.

All 2OMePS oligomers were also examined at 1 µM concentration in the normal human myoblasts. Two days after transfection, the cells were harvested and hE50 skipping was examined by RT-PCR and Nested-PCR. Gel analysis demonstrated transcripts corresponding to dystrophin mRNA with hE50 skipped in the cells treated with the following 7 AOs, hE50AO4PS, hE50AO5PS, hE50AO6PS, hE50AO12PS, hE50AO13PS, hE50AO22PS and hE50AO23PS (Table 1). Three of them, hE50AO5PS, hE50AO12PS and hE50AO23PS revealed strongest bands of RT-PCR and Nested-PCR products representing hE50 skipping with the efficiencies up to 29%, 25% and 65% respectively (Figure 2A). The overall highest levels of skipping efficiency were detected with hE50AO23PS and confirmed by repeated (3 times) experiments.

We also examined 7 PMOs in the human myoblasts (Table 1). The efficiency of exon skipping was similar to that obtained by the equivalent 2OMePS AOs at the same 1 µM concentration. The hE50AO5PMO and hE50AO12PMO were effective for specific hE50 skipping at approximately 28% and 45% rate respectively, whereas no hE50 skipping was detected with the hE50AO14PMO, hE50AO26PMO and hE50AO27PMO (Table 1). The highest efficiency for hE50 skipping was again detected in the cells treated with hE50AO23PMO, accounting for approximately 85% of the total amplified mRNA signal (Figure 2B). The specificity of the hE50 skipping was confirmed by sequencing of the bands with the expected size (Figure 2C).

### Targeting human dystrophin exon 50 in DMD-patient-derived skin fibroblasts

We next examined the 7 PMO oligomers for hE50 skipping in a DMD–patient-derived skin fibroblasts (Table 1). The cells contained the deletion of human dystrophin exon 51 resulting in a shift in reading frame. Further skipping of the hE50 would restore the reading frame of the dystrophin mRNA. The expression of dystrophin mRNA in the fibroblasts was confirmed by RT-PCR and Nested-PCR. The cells were treated with each oligomer at the same 1 µM concentration. RT-PCR and Nested-PCR was used to identify hE50 skipping 3 days after transfection. Four oligomers demonstrated clearly detectable hE50 skipping (Table 1). The 3 oligos, hE50AO12PMO, hE50AO5PMO and hE50AO23PMO produced hE50 skipping with the efficiency up to 25%, 34% and 62% respectively in repeated experiments (Figure 2D).

### Dose dependent human dystrophin exon 50 skipping with the hE50AO23PMO

**Figure 3:**
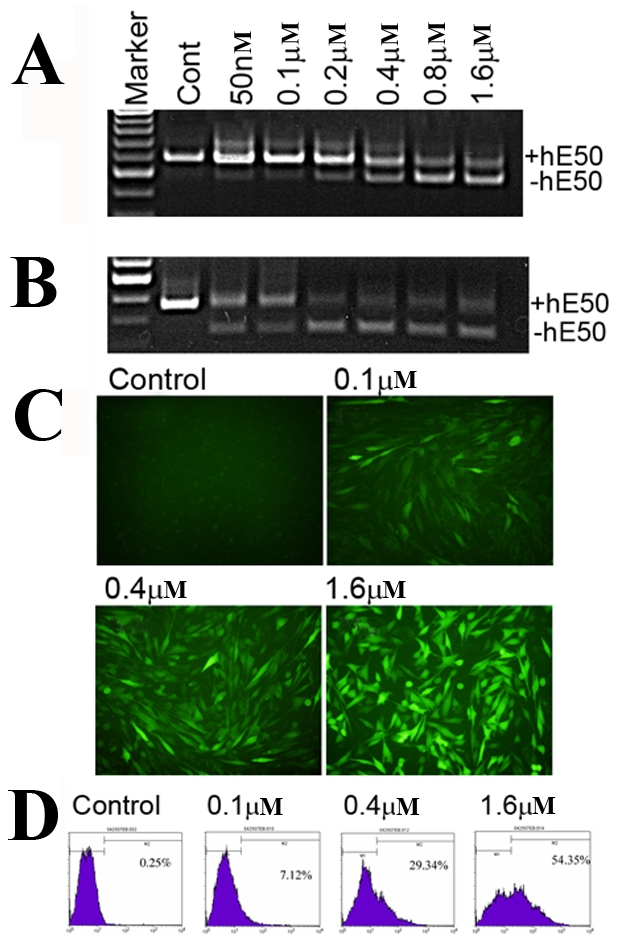
Dose dependent human dystrophin exon 50 skipping with the hE50AO23PMO in normal human myoblast and skin fibroblasts of a DMD patient with exon 51 deletion. The signal intensity of the strongest bands representing exon 50 skipping was 71% and 65% in the normal human myoblas (A) and skin fibroblasts (B) respectively (measured with NIH ImagJ). (C). C2C12hE50 reporter myoblasts. (D). FACS analysis of the C2C12hE50 reporter cells treated with the hE50AO23PMO with the concentrations specified.

We also further examined the dose response of the hE50AO23PMO with the highest efficiency as a potential candidate for further drug development. A dose-dependent increase in hE50 skipping was observed in all the three cell culture systems. The efficiency of hE50 skipping was detectable at 50 nM and increased to approximately 70% at 1.6 µM in the normal human myoblasts (Figure 3A). Skipping efficiency also reached 64% in the DMD patient derived skin fibroblasts at 0.2 µM concentration. However, skipping efficiency did not increase significantly further when more than 0.2 µM concentrations were used (Figure 3B). In the C2C12hE50 cells, the enhanced exon skipping with increasing doses of hE50AO24PMO exhibited as both increased number of GFP positive cells and the intensity of the GFP signals within the cells (Figure 3C). Quantitative flow cytometry analysis further confirmed the dose-dependent inductions of GFP expression by the hE50AO24PMO, with up to 54% of the cells expressing GFP at 1.6 µM concentration (Figure 3D).

### Vivo-PMO induced effective dystrophin exon skipping in muscles of normal C57 mouse

**Figure 4:**
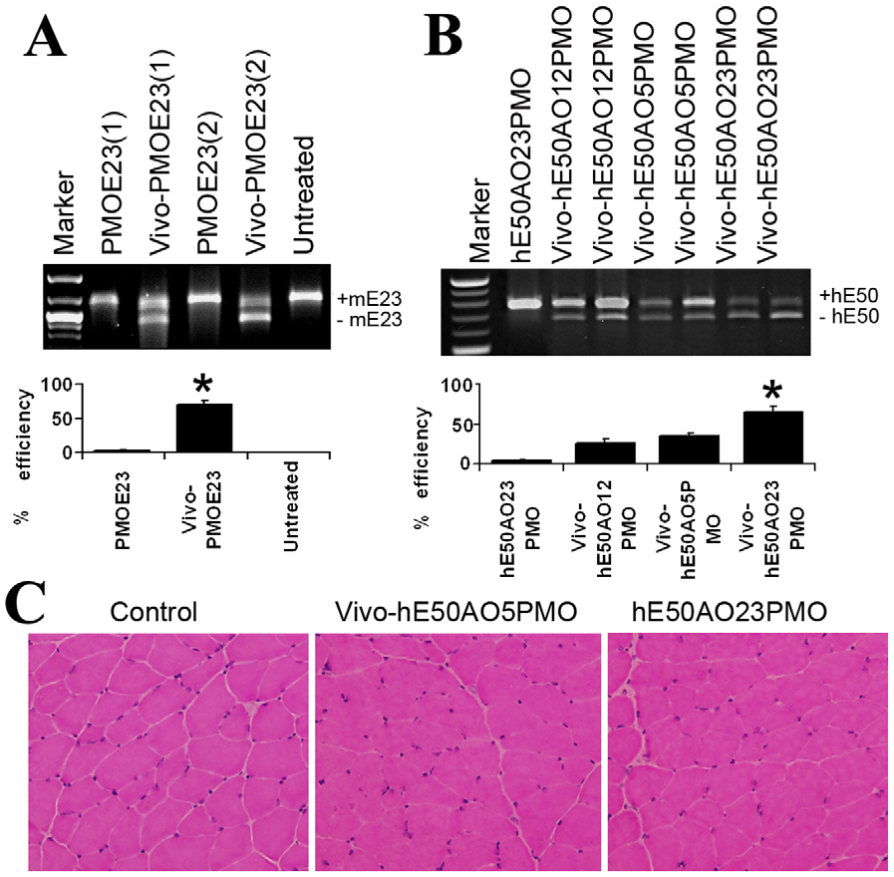
Effect of Vivo-PMO for dystrophin exon skipping in tibialis anterior (TA) muscles of normal C57 mice and in the hDMD/*mdx* mice. (A). The signal intensity of the bands representing mouse dystrophin exon 23 skipping with Vivo-PMOE23 from normal C57 mice was 70±7%. (B). Signal intensity of the bands representing human dystrophin exon 50 skipping after Vivo-hE50AO23PMO treatment from the hDMD/*mdx* mice was 65±7%, significantly higher when compared with 25±6%, 34±4% detected after Vivo-hE50AO12PMO and Vivo-hE50AO5PMO treatment. Results from 2 TA muscles for each Vivo-PMO are presented. Signal intensity was measured with NIH Image J. Results are presented as mean values ± SE with statistical analysis (t-test). *P<0.01 was obtained by comparison of the group(s) with PMOE23 and hE50AO12PMO group in (A) and (B) respectively (n = 5). (C). H&E staining of the TA muscles treated with Vivo-PMOs. No change in muscle histology was detected. Control, saline injected TA muscle.

We recently demonstrated that PMOs tagged with arginin-rich peptide (PPMO) and octa-guanidine (Vivo-PMO) were able to target apparently normal muscles for exon skipping systemically [7], [8]. We therefore first tested the Vivo-PMOE23 (+07–18) for targeted skipping of mouse dystrophin exon 23 in the normal C57 mice, so the results can be compared to the exon skipping efficiency of the same AO in the dystrophic *mdx* mice [8]. TA muscles of the C57 mice were injected with 10 µg Vivo-PMOE23 or PMOE23 and examined for the exon23 skipping 2 weeks later. High levels (approximately 40–70%) of exon 23 skipping were detected by RT-PCR (without Nested-PCR) in the Vivo-PMOE23 treated muscles whereas skipping was not detectable in the muscles treated with PMOE23 (Figure 4A). The levels of exon skipping efficiency were therefore similar to that achieved in the dystrophic *mdx* mice [8]. This result confirmed that Vivo-PMO is able to transfect the normal muscle and induce targeted dystrophin exon skipping with similar efficiency to that in dystrophic muscles. As expected, no difference in levels of dystrophin protein was observed in the muscles between treated and control C57 mice, due to the fact that the truncated mRNA will be translated into functional dystrophin protein [4].

### Vivo-PMO induced human dystrophin exon 50 skipping in the hDMD/*mdx* mice

It is desirable to test the selected AOs targeting human dystrophin exons in animal models for efficacy as part of AO drug development. The only available model is the humanized *mdx* mouse (hDMD/*mdx*) [25], [31]. To test whether effective skipping of human dystrophin exon can be achieved in the hDMD/*mdx* mice, we first examined 3 most effective PMOs as Vivo-PMOs, the hE50AO12PMO, Vivo-hE50AO5PMO and Vivo-hE50AO23PMO for hE50 skipping in comparison with unmodified hE50AO23PMO. Each TA muscle from the mice aged 5–7 weeks received total of 10 µg PMO or Vivo-PMO by intramuscular injection and was examined for hE50 skipping by RT-PCR (without further nested PCR amplification) 5 days later. Human dystrophin exon 50 skipping was not clearly detected in the muscles treated with the hE50AO23PMO (Figure 4B). However, the efficiency of exon skipping reached 25±6%, 34±4% and 65±7% (n = 5) with Vivo-hE50AO12PMO, Vivo-hE50AO5PMO and Vivo-hE50AO23PMO respectively (Figure 4B). Specific skipping of hE50 was confirmed by subsequent sequencing. No increases in muscles damage, inflammatory cellular infiltrations, or necrotic fibers were observed in the muscles treated by any of the Vivo-PMOs when compared to the PMO (Figure 4C). These results suggest that Vivo-PMOs can achieve high levels of human dystrophin exon skipping in the muscle of the hDMD/*mdx* mice without notexin pre-treatment which has been required for inducing human dystrophin exon skipping with untagged PMO and 2OMePS [26].

### Systemic delivery of Vivo-PMOs produced high levels of human dystrophin exon 50 skipping in both skeletal and cardiac muscles

**Figure 5:**
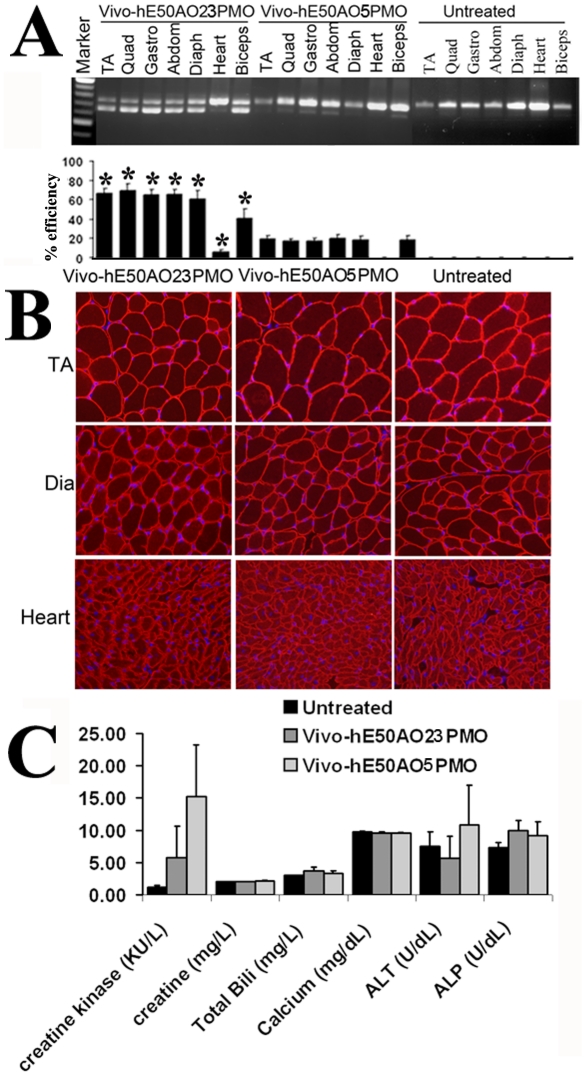
Effect of Vivo-PMO for human dystrophin exon 50 skipping systemically in the hDMD/*mdx* mice. TA, tibialis anterior; Quad, quadriceps; Gastro, gastronemium; Abdom, abdominal; Diaph, diaphragm. The signal intensity of the bands representing human dystrophin exon 50 skipping was 70±13% and 5±1%, 20±5% and 0% in body-wide skeletal muscles and cardiac muscles treated with Vivo-hE50AO23PMO and Vivo-hE50AO5PMO respectively (measured with NIH ImagJ) (A). Results are presented as mean values ± SE with statistical analysis (t-test). *P<0.01 was obtained by comparison of the Vivo-hE50AO23PMO group with the hE50AO5PMO group. (n = 5) (B). Immunohistochemical staining shows similar intensity for dystrophin after the Vivo-PMO treatments in all muscles tested. (C). Serum enzyme tests show a slight increase in the levels of creatine kinase in the mice treated with Vivo-PMOs.

We then investigated systemic effects of the two most effective Vivo-PMOs from the local injection since therapeutic value of AOs for DMD patients relies on systemic treatment. The Vivo-hE50AO23PMO and Vivo-hE50AO5PMO at the dose of 15 mg/kg were injected intravenously into adult (5–7 weeks) hDMD/*mdx* mice. Both Vivo-PMOs induced clearly detectable hE50 skipping by RT-PCR (without Nested-PCR). Vivo-hE50AO23PMO induced more than 70±13% skipping efficiency in body-wide skeletal muscles tested and about 5±1% in cardiac muscles, significantly higher than the Vivo-hE50AO5PMO which showed less than 20±5% efficiency in all skeletal muscles, but no detectable exon skipping in the cardiac muscle (Figure 5A). All the mice treated with Vivo-PMOs had no weight change when compared to the controls treated with saline. There was no death during the treatment period. No clear changes in the levels of dystrophin expression were detected with immunostaining and western blot in the muscles including heart and diaphragm (Figure 5B). However, an increase in serum creatine kinase level was observed in the mice treated with the Vivo-PMOs (Figure 5C), suggesting a low level disturbance in membrane integrity possibly due to the slight reduction in dystrophin protein in some muscles as the mouse expresses the dystrophin only from the human dystrophin gene. The levels of other serum enzymes including ALT in the Vivo-PMOs-treated mice remained similar to those in the control mice (Figure 5C). Consistently no changes were observed in the muscles, liver and kidney by histology.

## Discussion

More than 1 and half decades ago, Kole and his colleagues first introduced AOs as splicing regulators for correcting inherited genetic disorder, Thalassemia. By blocking the aberrant splice sites sterically in the mutated β-globin gene, specific AO was able to restore the normal splicing pattern of the gene [28]. Since then, the same principle has been developed into a highly promising experimental therapy for the DMD. For this application, AOs are used as the drugs for skipping targeted dystrophin exons to restore the reading frame, thus the expression of functional proteins. Given that AO-mediated exon skipping is principally a drug therapy, one of the most critical factors for high efficacy of the remedy is the identification of effective AOs. Ideally, AO drugs targeting specific human dystrophin exon should be selected first in cell culture systems representing the targeted cells in human and the efficacy of the selected AOs will then be validated in vivo in animal models before the selected AOs are considered for further drug development including expensive toxicology and pharmacology tests. These requirements however have been difficult to meet as human muscle fibers can not be maintained in culture long enough for AO tests and no dystrophic animal models containing human dystrophin gene with specific DMD mutations exist. Currently the most widely used model for AO selection have been the human myoblast culture system. The relevance of AOs selected through the cultures to the efficiency in vivo has been to a certain extent supported with the AOs targeting mouse dystrophin exon 23 and human dystrophin exon 51 in the *mdx* mice and in the afore mentioned clinic trials [16]. These results suggest that the majority of AOs selected in the myoblast culture is likely to have the potential to skip the targeted exon in vivo. However, discrepancy in skipping efficiency between in cell culture and in vivo in animal models with same AO has been reported. In the dystrophic dog model, one highly effective AO selected in culture of the dog's myoblasts was later found to be of limited exon skipping efficacy in vivo [10]. This raises the concern that selected AO through myoblast cultures alone may fail to achieve expected efficiency in muscle fibers in vivo for targeted exons, a risk too costly to be ignored. In this study, we also observed discrepancy in efficiency of exon skipping between cell cultures and the hDMD/*mdx* mice in vivo with some AOs. While the hE50AO23PMO consistently induced highest hE50 skipping in all cell culture systems and in the hDMD/*mdx* mice in vivo, the relative efficiency of hE50 skipping with hE50AO12PMO and hE50AO5PMO varies in different systems. The hE50AO12PMO induced clearly higher efficiency in the human myoblasts than hE50AO5PMO, whereas the hE50AO5PMO induced higher skipping efficiency in the reporter cells, DMD-derived fibroblasts and in the muscles of hDMD/*mdx* mice. Although discrepancy in exon skipping efficiency in different testing systems is not a common feature for most AOs tested in this study, our finding further suggest that caution needs to be excised to minimize the risk of selecting AO with lower efficiency in vivo for antisense drug development.

One limitation for using myoblast culture to select AO is the relative low sensitivity of the system to distinguish AOs with similar exon skipping efficiency, but the difference between such AOs could be significant when applied to the patients in clinics. This is because evaluation of targeted exon skipping efficiency in human myoblasts as well as in fibroblasts relies almost exclusively on second round Nested-PCR, which together with the initial RT-PCR procedure, has a degree of variation to determine the relative amount of transcripts from different samples. It is therefore apparently insufficient to select AO for further drug development based on Nested-PCR alone in myoblast culture and desirable to search for culture systems with higher sensitivity and better quantification to evaluate exon skipping efficiency [32]. Confirmation of AO with high efficiency for specific exon skipping in additional screening system would also suggest a higher probability for the selected AO to be effective in wider cellular environments, thus in human muscles of different DMD patients in vivo. Here we explored the use of a GFP reporter myoblast culture system for screening AOs targeting human dystrophin exon. The system was originally established by Kole and his colleagues to identify AO sequences for effective blocking of the aberrant splicing site in the mutated β-globin gene [28]. We found that the reporter system is more sensitive for identifying AOs capable of skipping human dystrophin exon 50 than the human myoblast culture. The exon skipping efficiency of individual AO demonstrated in the reporter cells is in general consistent to that observed in the human myoblasts. More importantly, the GFP reporter system provides direct visual presentation under microscope for the targeted exon skipping and quantitative measurement of the skipping efficiency. These together with the fact that chimeric GFP/hE50 RNA can be stably expressed at high levels provide greater capability to distinguish the differential antisense effect with individual AOs. Thus, although the reporter system cannot replace the myoblast cultures for AO screening, the advantages make it a valuable additional screening system. A combined use of the GFP-based reporter with human myoblast cultures and cells from applicable DMD patients would be better suited for selection of AOs targeting human dystrophin exon in vitro.

Traditional drug development requires validation of the efficacy in animal models of same disease entity. Antisense therapy for DMD uses AOs complementary to human dystrophin RNA. Therefore, animal models in general are not useful for efficacy validation due to species specificity of gene sequence. This poses a significant hurdle for AO drug development as drug developers will be unable to validate the efficacy of AO targeting human dystrophin exon in animal models, which is generally considered to be essential for regulatory approval of clinic trials. To mitigate the problem, van Deutekom's group in Netherlands created a transgenic mouse containing the entire human dystrophin gene [25]. A human dystrophin gene transgenic *mdx* mouse was also produced by crossbreeding with the *mdx* mouse (hDMD/*mdx*). However, expression of the normal human dystrophin compensates for the deficiency in mouse dystrophin protein. The hDMD/*mdx* mouse therefore is phenotypically normal, which severely limits the delivery efficiency of AOs of both chemistries, 2OMePS and PMO [26], [29]. Limited skipping of human dystrophin exon in the mouse had so far been achieved in local muscles only after notexin pretreatment [26]. However, we and others recently reported that the PPMO and Vivo-PMO were able to deliver the PMO oligomers effectively into near 100% of skeletal and cardiac muscles of the *mdx* mice without notexin pretreatment [7], [8]. Since the cardiac muscle and a proportion of skeletal muscle fibers in the mouse are of limited pathology, the results suggest that the tagged PMOs could be used to target apparently normal muscles for exon skipping systemically. This notion was first proved in the C57 normal mice with the Vivo-PMOE23 targeting mouse exon 23. Up to 70% efficiency in exon 23 skipping was achieved in the TA muscles when examined by RT-PCR. This efficiency at the mRNA level is similar to that observed in the dystrophic *mdx* mice with the same Vivo-PMO. This result together with the demonstration that similar efficiency can also be achieved for skipping human dystrophin exon 50 in the hDMD/*mdx* mouse clearly indicate that the hDMD/*mdx* mouse becomes a valuable model for testing AOs targeting human dystrophin when Vivo-PMO is used. Furthermore, systemic efficiency established with the known AOs in vivo, such as that with PMOE23 targeting mouse exon 23 could be used as a reference to assess the potential efficacy of AO targeting human dystrophin exon in vivo. With the efficacy of AOs targeting human dystrophin exon 51 may soon become available, systemic efficiency of any newly selected AOs can then be compared directly to that of the AO targeting exon 51 in the hDMD/*mdx* mice. Different dose regimens may also be formulated to assess desirable therapeutic effect systemically. This is especially useful for antisense therapy to DMD as many AO drugs are required for the treatment of majority of DMD boys.

In summary, our results demonstrate that a GFP-based reporter myoblast culture is a sensitive screening system for selection of AOs targeting human dystrophin exons. The use of Vivo-PMO enables AOs targeting human dystrophin exons to be examined in the hDMD/*mdx* mouse in vivo systemically, which is essential for drug development and regulatory approval. The effective hE50AO23 sequence identified in this study has now been further validated and selected by AVI Biopharma for further drug development targeting hE50. AOs selected by a combination of in vitro and in vivo tests are likely more robust to provide a high efficacy in patients.
